# Supplementary material for: Seminoma and Embryonal Carcinoma Footprints Identified by Analysis of Integrated Genome-Wide Epigenetic and Expression Profiles of Germ Cell Cancer Cell Lines
Source: PLoS One. 2014 Jun 2;9(6):e98330. doi: 10.1371/journal.pone.0098330 (PMC4041891; doi:10.1371/journal.pone.0098330)
Supplement: File S1 — ZIP file containing DMRforPairs output for significant regions. Please start from the html files. (ZIP) [file pone.0098330.s008.zip › figures/29047.pdf]

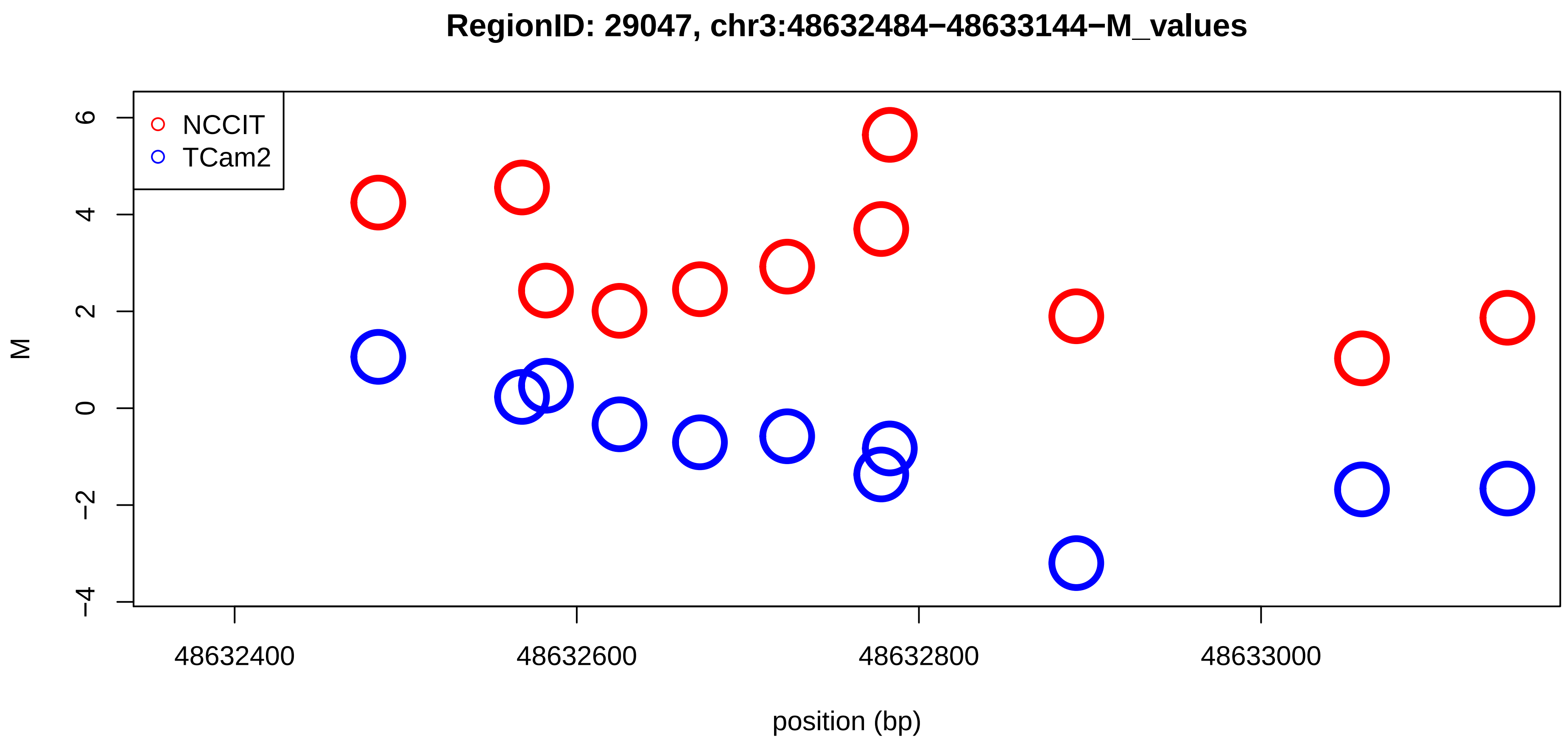

RegionID: 29047, chr3:48632484−48633144−M_values
NCCIT
TCam2
M
6
4
2
0
−2
−4
48632400
48632600
48632800
48633000
position (bp)

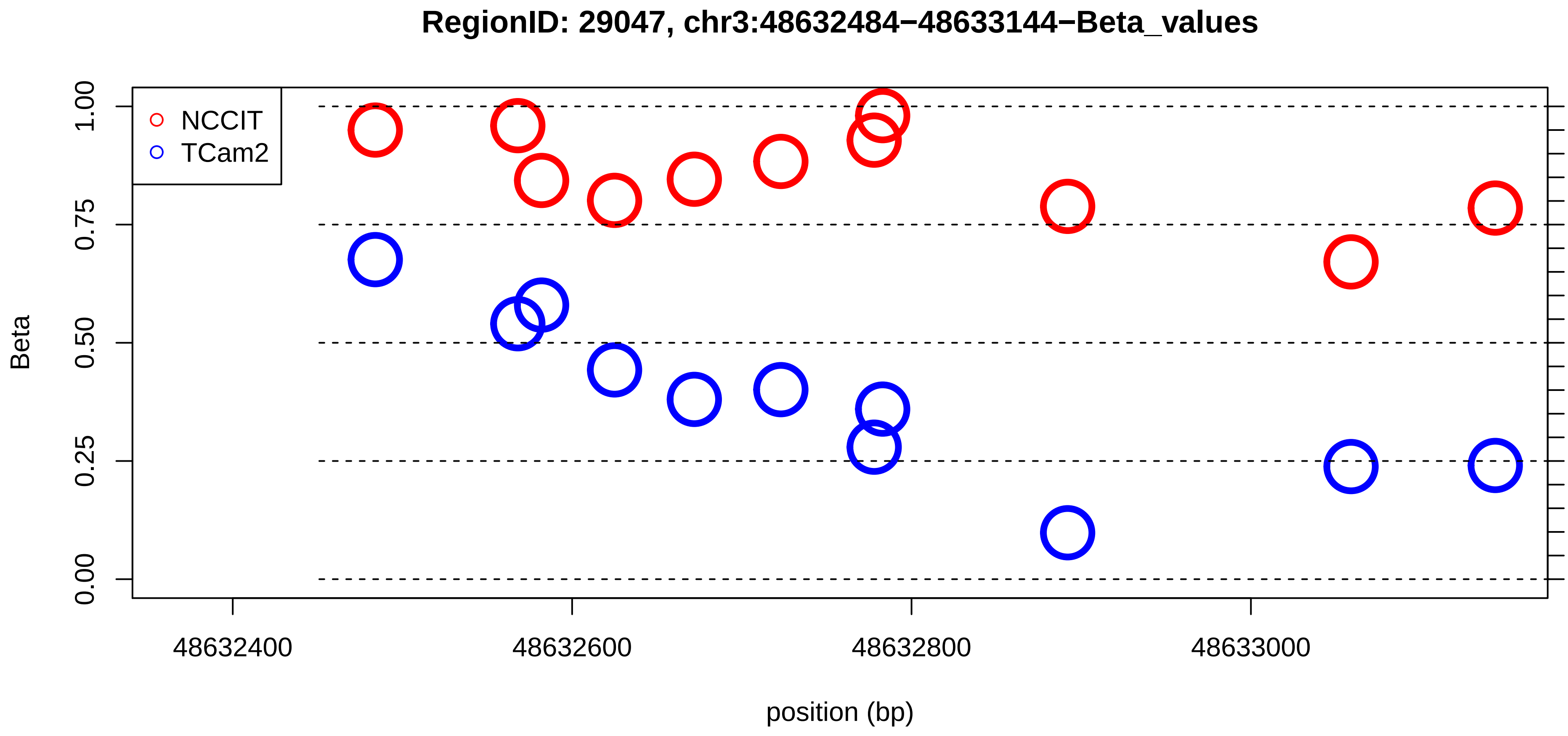

RegionID: 29047, chr3:48632484−48633144−Beta_values
NCCIT
TCam2
Beta
1.00
0.75
0.50
0.25
0.00
48632400
48632600
48632800
48633000
position (bp)

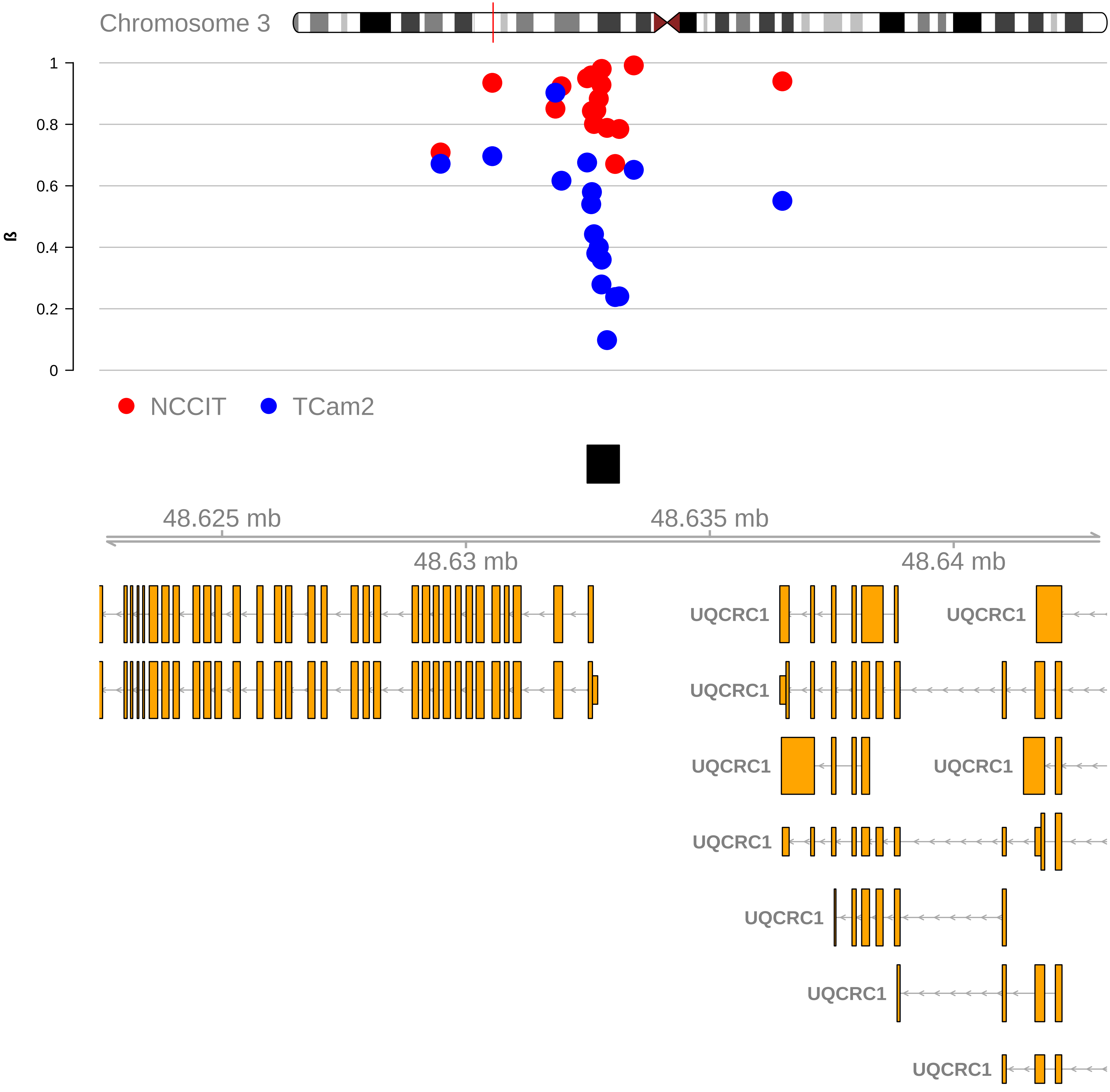

Chromosome 3
ß
1
0.8
0.6
0.4
0.2
0
NCCIT
TCam2
48.625 mb
48.635 mb
48.63 mb
48.64 mb
UQCRC1
UQCRC1
UQCRC1
UQCRC1
UQCRC1
UQCRC1
UQCRC1
UQCRC1
UQCRC1
